# Insight into live bird markets of Bangladesh: an overview of the dynamics of transmission of H5N1 and H9N2 avian influenza viruses

**DOI:** 10.1038/emi.2016.142

**Published:** 2017-03-08

**Authors:** Jasmine C M Turner, Mohammed M Feeroz, M Kamrul Hasan, Sharmin Akhtar, David Walker, Patrick Seiler, Subrata Barman, John Franks, Lisa Jones-Engel, Pamela McKenzie, Scott Krauss, Richard J Webby, Ghazi Kayali, Robert G Webster

**Affiliations:** 1 Department of Infectious Diseases, St Jude Children's Research Hospital, Memphis, TN 38105, USA; 2 Department of Zoology, Jahangirnagar University, Dhaka 1342, Bangladesh; 3 Department of Anthropology, University of Washington, Seattle, WA 98105, USA; 4 Department of Epidemiology, Human Genetics, and Environmental Sciences, University of Texas Health Sciences Center, Houston, TX 77459, USA; 5 Human Link, Hazmieh, Baabda 1107-2090, Lebanon

**Keywords:** Bangladesh, epidemiology, H5N1, H9N2, influenza, live bird market, poultry, surveillance

## Abstract

Highly pathogenic avian influenza (HPAI) H5N1 and low pathogenic avian influenza (LPAI) H9N2 viruses have been recognized as threats to public health in Bangladesh since 2007. Although live bird markets (LBMs) have been implicated in the transmission, dissemination, and circulation of these viruses, an in-depth analysis of the dynamics of avian transmission of H5N1 and H9N2 viruses at the human–animal interface has been lacking. Here we present and evaluate epidemiological findings from active surveillance conducted among poultry in various production sectors in Bangladesh from 2008 to 2016. Overall, the prevalence of avian influenza viruses (AIVs) in collected samples was 24%. Our data show that AIVs are more prevalent in domestic birds within LBMs (30.4%) than in farms (9.6%). Quail, chickens and ducks showed a high prevalence of AIVs (>20%). The vast majority of AIVs detected (99.7%) have come from apparently healthy birds and poultry drinking water served as a reservoir of AIVs with a prevalence of 32.5% in collected samples. HPAI H5N1 was more frequently detected in ducks while H9N2 was more common in chickens and quail. LBMs, particularly wholesale markets, have become a potential reservoir for various types of AIVs, including HPAI H5N1 and LPAI H9N2. The persistence of AIVs in LBMs is of great concern to public health, and this study highlights the importance of regularly reviewing and implementing infection control procedures as a means of reducing the exposure of the general public to AIVs.

## INTRODUCTION

Active surveillance is key to our understanding of influenza A viruses (IAVs) currently circulating in humans and animals throughout the world. Waterfowl are the natural reservoir of nearly all IAVs that have been detected in other avian and mammalian species.^1^ Avian influenza viruses (AIVs), replicate in the respiratory and/or digestive tracts of infected birds.^2^ Transmission of AIVs to domestic poultry naturally occurs at interfaces where wild birds and domestic poultry co-exist. AIVs are typically transmitted to humans as a result of exposure to infected domestic birds.^1^ Through active longitudinal surveillance of both domestic and wild birds, we can determine the prevalence of these viruses, but also obtain a better understanding of their transmission at the human–animal interface and develop strategies to mitigate the incidence of such transmission.

There have been many studies on the impact of the poultry industry on AIV transmission worldwide, giving rise to numerous recommendations for interventions.^3^, ^4^, ^5^, ^6^ However, in Bangladesh, a country with one of the highest human population densities, where people rely heavily on domestic fowl for both sustenance and income, little is known about what relative effect the live poultry industry has on both the currently circulating strains of AIVs and the risk to the general public. Active surveillance involving the repeat collection of hundreds of samples from poultry and the environment in live bird markets (LBMs) and farms on a monthly basis has enabled infections and outbreaks in Bangladesh to be closely monitored. Since 2008, several different subtypes of low pathogenic avian influenza (LPAI) viruses have been isolated from LBMs and farms in Bangladesh, with H9N2 being the predominant subtype. Highly pathogenic avian influenza (HPAI) H5N1 is the second predominant subtype found in LBMs and farms in Bangladesh, despite the use of anti H5 vaccines.^7^, ^8^ With both HPAI H5N1 and LPAI H9N2 currently circulating in LBMs all year round,^9^ the co-infection of market poultry populations with HPAI H5N1 and LPAI viruses, particularly H9N2, is of great concern. Reassortment of these LPAI viruses with HPAI H5N1 could produce novel influenza viruses capable of crossing the interspecies barrier and causing zoonotic transmission and infection in humans.

As of January 2016, three cases of human infection with H9N2 viruses had been reported from Bangladesh.^10^ The most recent case involved a poultry market worker who handled sick poultry prior to the onset of symptoms. Previous studies in Bangladesh have shown that all H9N2 viruses isolated possess the L226 mutation in the receptor binding pocket of the HA1 protein that is known to confer specificity to the human-like α-2,6 sialic acid linked cell receptors.^11^ H9N2 subtype was isolated all year round primarily from chickens, and showed antigenic similarity to a human H9N2 virus isolated in Bangladesh in 2011. Those viruses evolved from a prototype G1 clade virus to acquire mammalian host-specific mutations in the internal genes.^12^ No molecular markers were found in the NA gene to confer resistance to antiviral drugs; however, the M2 proteins showed substitutions that confer resistance to M2-blocker antiviral drugs such as Amantadine. Although the total number of reported cases of H9N2 human infection in Bangladesh is low, the ability of these viruses to mutate rapidly and acquire markers for mammalian host adaption highlights the critical importance of continued surveillance in this country.

Two clades of H5N1 viruses were in circulation in Bangladesh from 2008 to 2016. H5N1 clade 2.2.2 circulated from 2007 to 2011, while clade 2.3.2.1a circulated from 2011 to present.^13^ Recent studies have shown that both clades of H5N1 viruses in Bangladesh have HA residues that confer both increased virus binding to α-2,6 receptors and a preference for α-2,3-linked sialic acid for greater avian infectivity.^13^ It has been reported^9^ that the only clade of H5N1 detected since 2011 was 2.3.2.1a, and seasonality of H5N1 infection in birds in LBMs was inapparent because it was isolated every month except in August 2013. Recently isolated H5N1 viruses from LBMs have diverged as a result of local intra- and inter-clade reassortment. This suggests that poultry trade and LBMs, rather than virus introduction from outside of the country contribute to the perpetuation of a genetically stable H5N1 lineage.^9^

As of July 2016, Bangladesh had reported eight human infections with H5N1 since 2003, one of which was fatal.^14^ In most of these and other cases of H5N1 human infection in endemic countries, there is a link between direct or close contact with infected or dead poultry and the onset of disease symptoms.^15^ Despite the pathogenicity of H5N1 viruses in SPF birds, infected poultry occasionally lack disease symptoms making it difficult to diagnose influenza infection in the LBMs. Cross-protection by H9N2 co-circulating with H5N1 remains a plausible hypothesis to explain the lack of morbidity and mortality of poultry in LBMs.^9^ Given human population density, popularity of LBMs in Bangladesh, and the co-circulation of HPAI H5N1 and LPAI viruses throughout the year, there is a risk of transmission to and infection of a large number of people in the event of an AIV pandemic.

Here, we report epidemiological findings as a result of active surveillance in LBMs and poultry farms in Bangladesh from November 2008 to February 2016 in order to acquire a more comprehensive understanding of the dynamics of AIV transmission and infection within the poultry industry in Bangladesh, and to recommend mitigating measures to reduce the potential pandemic threat to public health.

## MATERIALS AND METHODS

### Avian influenza surveillance

Active surveillance for AIVs in poultry in Bangladesh has been ongoing since November 2008. Each month, water, fecal, oropharyngeal and cloacal samples were collected from birds in retail markets, wholesale markets, backyard flocks and poultry farms as previously described.^7^ Wholesale markets were the initial collection point for birds entering the market system. Approximately 5000–10 000 birds were sold each day from five wholesale markets that service Dhaka and surrounding areas. Of the five wholesale markets present, sample collection during this time took place at two conveniently selected markets. Although some birds were available for consumer purchase within wholesale markets where no slaughtering took place, these birds were mainly distributed to various retail markets throughout Dhaka. In this study, retail markets were defined as those places where consumers can go to purchase live poultry for slaughter. Approximately 500–1000 birds were sold each day from stalls and shops at a total of 50 retail markets. Samples were collected from eight different retail markets during the course of this study. Birds for meat consumption were slaughtered within designated areas in the retail markets.

Backyard flocks were defined as groups as few as 10 to as many as 100 birds, which reside in free-range conditions during the day where they are allowed to scavenge and intermingle with wild birds. These birds were typically housed in enclosures overnight, and were supplied to wholesalers by local villagers or farmers who specialized in poultry rearing. Samples were collected from nine conveniently selected backyard flocks. The term ‘Farm' was used to describe commercial farms with more than 100 birds that specialized in raising poultry for eggs and/or meat. Samples were collected from 11 farms throughout Bangladesh. While the birds in commercial farms were not allowed to mingle with wild birds directly and were confined to a limited area, there was still low biosecurity and a risk for indirect contact with wild birds and interspecies transmission of AIVs.

### Sample collection

Samples collected were placed in PBS/glycerol isolation media and stored at −80 °C until an initial screening was performed. The total number of samples collected ranged from 300 to 600 each month from November 2008 to May 2012. Samples included oropharyngeal, cloacal and fecal swabs as well as water samples from drinking water troughs. Due to limited funds, the number of samples collected decreased to ~210 from November 2012 to the present. Epidemiological data of 21 096 samples collected from domestic poultry in backyards, farms and LBMs between November 2008 and February 2016 were analyzed. Field data obtained consisting of swab date, sample/host ID number, host common name, bird behavior, domestic bird characterization, bird health status, sample type, age, sex and capture status were used for the analyses conducted in this study. Bird movement patterns were used to categorize the subjects into the following categories: wild migrating birds, wild non-migrating birds, domestic poultry and wild unknown birds. The domestic bird characterization was used to differentiate between retail birds that were sampled at LBMs and farm birds, which were birds that had not been sold into the market system. This study was approved by the Institutional Animal Care and Use Committee at St Jude Children's Research Hospital (Memphis, TN, USA).

### Sample screening

All samples collected were screened in the biological safety level 3+ laboratory of the Center of Excellence for Influenza Research and Surveillance (CEIRS) at St Jude Children's Research Hospital. Both virology and molecular biology techniques were utilized for sample screening. In 2008, all swabs collected were inoculated into 10-day-old embryonated chicken eggs and incubated at 35 °C for 72 h. Eggs were then chilled at 4 °C overnight and allantoic fluid harvested to test for influenza infection via hemagglutination assay (HA). HA was performed using 0.5% chicken erythrocytes according to WHO protocol. Samples that yielded HA titers >1 were subsequently subtyped via hemagglutination inhibition (HI) assay and/or Sanger sequencing. Real-time RT-PCR (rRT-PCR) was introduced shortly after the start of surveillance (2009) as a means to decrease processing time. Viral RNA was extracted from the original swabs collected and processed via rRT-PCR as previously described.^11^ During the 2009–2012 surveillance period, all samples that tested positive for influenza matrix gene by rRT-PCR were injected into eggs for virus isolation.

This strategy of using both molecular and virology techniques consecutively for surveillance remained consistent until 2013. In 2013, influenza matrix gene rRT-PCR positive samples were rescreened using H5-specific primers and probe to test for the presence or absence of HPAI H5N1 via rRT-PCR. All rRT-PCR H5-positive samples were injected into eggs, whereas only 10% of non-H5 rRT-PCR positive samples were injected into eggs. The selection of non-H5 rRT-PCR samples was determined based on matrix gene rRT-PCR cycle threshold (Ct) value, species, location and sample type. The definition of rRT-PCR positivity was established based on 10-fold serial dilutions of control RNA down to approximately one copy number. The Ct value of the highest dilution of the controls was determined to be the limit of detection and served as the threshold for determining whether samples were influenza A positive or negative.

For the purposes of this study, any sample that was confirmed as having influenza A by rRT-PCR, HI assay, or sequencing was considered positive. Newcastle disease virus (NDV) was frequently found as co-infections with IAVs, including both velogenic and lentogenic strains. Samples subtyped as NDV or any other paramyxoviruses were not included in analyses unless influenza A virus subtypes had also been identified within the sample.

### Statistical analysis

Chi-square and Fisher's exact tests were used for the statistical comparisons. A *P*-value <0.05 was considered to indicate statistical significance. Analysis was performed using SPSS v18 (IBM, Armonk, NY, USA).

## RESULTS

Distribution of the collected samples and the frequency of influenza A detection within each category are shown in Table 1. Overall, influenza A was detected in 5060 of the 21 096 samples analyzed (24%). Of the 21 096 samples analyzed, 8081 (38%) were oropharyngeal, 4609 (22%) cloacal, 6395 (30%) fecal and 2011 (10%) water samples from drinking water troughs. Influenza A was most frequently detected in the water samples and oropharyngeal swabs (33%), and less frequently in cloacal swabs (19%), and fecal samples (14% *P*<0.001). The positive drinking water troughs samples came from markets with chickens (80%), quail (15%), and ducks (5% data not shown). The majority of the samples were from chickens (14 213 (67%)), followed by ducks (3426 (16%)), quail (2234 (11%)), pigeons (1165 (6%)) and geese (58 (0.3%)). The highest frequency of influenza detection came from quail (34%), followed by ducks (25%), chickens (23%), geese (19%) and pigeons (15%). The difference in detection by species was statistically significant (*P*<0.001). Around 21 000 samples (99%) were from domestic poultry for human consumption and the remaining were from bird species sold in LBMs as pets. The frequency of influenza detection in poultry intended for consumption was twice that in pets (24% vs 12%, *P* <0.001). Almost all samples (99%) were obtained from healthy birds and influenza A virus was detected in 24% of these.

By location, most samples were collected from LBMs (14 669 (70%)), farms (6202 (29%)) and backyard flocks (225 (1%)) (Table 1). Detection rates were 2%, 10% and 30% in backyard flocks, farms and LBMs, respectively, and the difference was statistically significant (*P*<0.001). Within the LBMs, 76% of the samples (11 137) came from retail LBMs as compared to 24% (3532) from wholesale LBMs. Detection rates were close, but the difference was statistically significant (30% in retail vs 32% in wholesale, *P*=0.027; Table 1).

**Figure 1:**
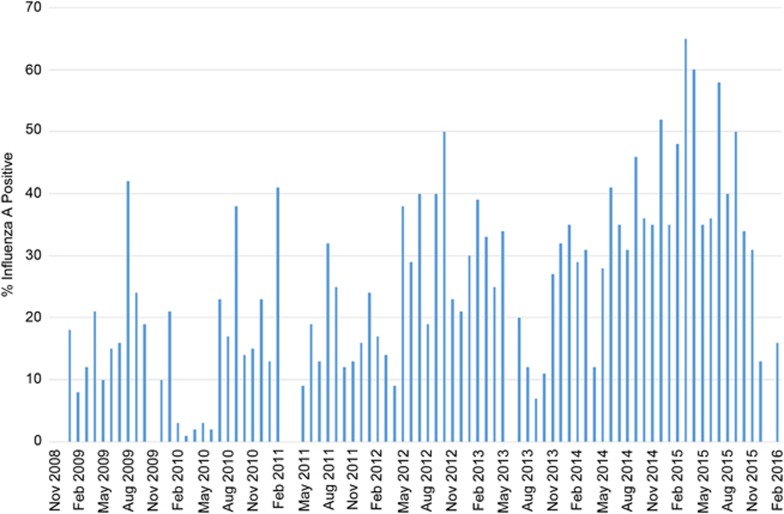
The percentage of influenza A-positive samples by month of each year of surveillance in Bangladesh. Bar graph shows the percentage of samples that were confirmed as influenza A positive for each surveillance year. All influenza A subtypes isolated are represented in this graph.

IAV detection increased during the course of this study. There was a noticeable increase in the detection frequencies in the latter years (2012–2016) compared to the earlier years of the study (2009–2011; Figure 1). Overall, no clear seasonal distribution of influenza A detection was observed as influenza A was continually detected at high rates each month of surveillance (Supplementary Figure S1).

For the different subtypes of IAVs in circulation in Bangladesh (Table 2), of the 5060 positive IAV samples, 1302 (26%) were subtyped, only 108 (8%) of the virus isolates were highly pathogenic H5N1 viruses, 1017 (78%) were low pathogenic H9N2 viruses, 133 (10%) represented co-infections between H5 and H9 viruses and 44 (3%) were other LPAI subtypes. The other subtypes included hemagglutinin subtypes H1, H3 through H7, H10 and H15 in various combinations with neuraminidase subtypes N1 through N3 and N5 through N9.

Table 2 shows the distribution of influenza A subtypes by study variables. We detected a statistically significant difference in the distribution of subtypes by sample type (*P*<0.001). H9N2 viruses were frequently detected in the oropharyngeal swabs (653/825, 79%) and water samples (134/158, 85%). These viruses were also detected in cloacal swabs (115/155, 74%) and fecal samples (115/164, 70%). Highly pathogenic H5 viruses were detected more frequently in cloacal and fecal samples (12% and 18%, respectively) than in the oropharyngeal and water samples (6% and 8%, respectively). H5/H9 co-infections were detected in all sample types at frequencies of 13%, 7%, 7% and 4% for oropharyngeal, cloacal, water and fecal samples, respectively. Other subtypes were sporadically detected in all sample types except water samples at frequency of 9% (fecal), 7% (cloacal) and 2% (oropharyngeal).

H9N2 viruses were detected at high frequencies in chickens, pigeons and quail (range 76%–100%). Of the samples from ducks, 9% tested positive for H9N2. Ducks had the highest detection rate for H5N1 viruses as 34% had the subtype. In all, 15% of quail samples and 4% of chicken samples tested positive for H5. H5/H9 co-infections were found mostly in ducks (23%) followed by chickens and quail (9% and 8%, respectively). Other subtypes were only detected in ducks. The difference in prevalence of subtypes by species was statistically significant (*P*<0.001). Nearly all subtypes were detected in healthy birds. H5, H9 and H5/H9 co-infections were significantly more prevalent (*P*<0.001) in LBMs than farms (9%, 80% and 11% vs 2%, 26% and 0%, respectively) while other subtypes were found mostly on farms (72%) rather than in LBMs (1%). Analysis within the LBM type revealed that H5, H5/H9 co-infections, and other subtypes were more prevalent in wholesale markets (25%, 15% and 3%, respectively) than retail markets (8%, 9% and 0.5%, respectively). H9N2 viruses were more common in retail markets (82%) than in wholesale markets (71%). Difference in subtype prevalence between market types was statistically significant.

**Figure 2:**
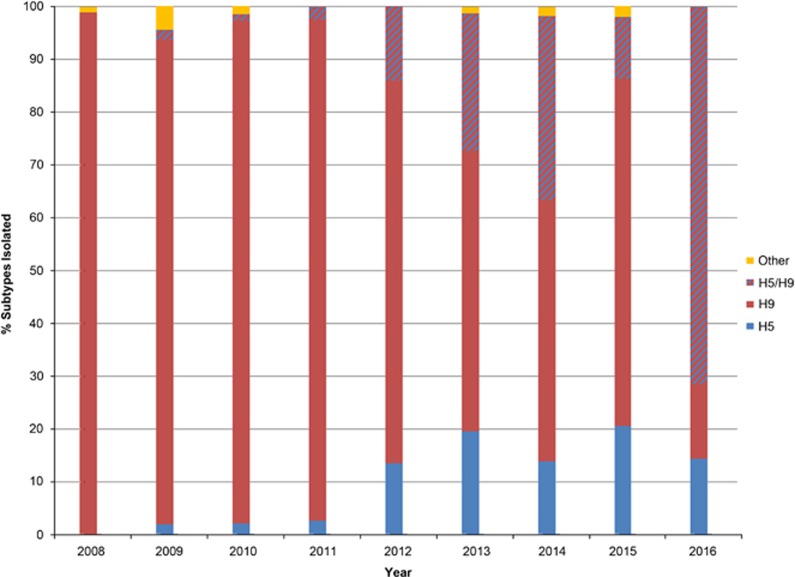
The percentage of subtypes isolated by year of surveillance in Bangladesh. Bar graph shows the percentage of samples that were isolated from eggs and subtyped each surveillance year. Samples that were isolated as co-infections of H5N1 and H9N2 are depicted with the red and blue striped bars. Other subtypes that were isolated include HA subtypes 1, 3–7, 10 and 15 in various combinations with NA subtypes 1–3 and 5–9.

Analysis of subtypes over time revealed that as of 2012, the frequency of H5 and H5/H9 detection increased (Figure 2). However, H9 remained the dominant subtype detected annually.

## DISCUSSION

Avian influenza has been a persistent problem and threat to public health in Bangladesh since 2007.^16^ Despite the fact that many studies have been conducted in countries such as China on the effect LBMs have on the dissemination of AIVs,^17^, ^18^, ^19^, ^20^ to date, there are no studies that provide in-depth analyses on the dynamics of transmission of avian influenza H5N1 and H9N2 viruses within live poultry markets in Bangladesh. The data presented here indicate that the drinking water serves as a reservoir of AIVs within LBMs. The similarity of the detection rate of AIVs in oropharyngeal samples and in water troughs suggests that the close proximity of poultry housed in LBMs, the shedding of H9N2 from the oral cavity and the sharing of the same water troughs facilitate the dissemination of AIVs. As quail are smaller allowing for more individuals to be caged together, more susceptible to influenza infection than chickens or ducks, and have been implicated in the land-based spread and adaptation of H9 viruses to other hosts,^21^ the results presented showing quail as the species with the highest detection of IAV infection are not surprising.

A large proportion of the poultry-to-poultry transmission events of HPAI H5N1 and LPAI H9N2 occur within the LBMs, and this is supported by the fact that the overwhelming majority of IAV infections were observed in domestic poultry intended for consumption. This is also supported by the data showing influenza A detection being twice as prevalent in domestic poultry compared to pet birds of the same species. Also, breeding and housing practices for pet birds in Bangladesh do not allow for interspecies interactions, which may also result in fewer AIV transmission events. In order to reduce the spread of AIVs within domestic poultry, focus should be placed on the implementation of infection control measures throughout the poultry production system with emphasis on LBMs. Because birds within LBMs rarely show any disease symptoms and nearly all influenza A isolates came from apparently healthy birds, reliance upon apparent health as a basis for classification of birds as infected with AIV or not may be misleading. It is imperative to place emphasis on market hygiene despite apparently healthy birds in order to prevent the spread of AIVs.

The low prevalence of AIVs in backyard flocks and on farms compared to LBMs suggests that housing birds in confined cages plays a major role in the propagation of IAVs. The low biosecurity in backyard flocks and commercial farms and the fact that all non-H5/H9 viruses were detected in ducks, which primarily enter the market system from backyard flocks, suggest that either direct or indirect contact with wild birds is the source of infection to backyard and farm birds. When infected birds arrive at LBMs, the viruses they shed become persistent and more prevalent. Despite that IAV detection in wholesale and retail markets were very similar, the differences between IAV detection are subtype-specific. Wholesale markets serve as a conduit for the amplification of H5N1, H5/H9 co-infections and other non-H5/H9 AIVs. Poultry that enter the LBM system through wholesale markets may be more susceptible to acquiring influenza infection, possibly due to crowded conditions, stress of transport, and being housed in a contaminated environment.

The presence of H5N1 and co-infections of H5/H9, along with non-H5/H9 viruses, in wholesale markets raises concern for public health. The data presented show that ducks are the primary species for H5N1, H5/H9 co-infections and non-H5-H9 infections suggesting within the LBMs, ducks are the potential reservoirs for reassortment events to take place. This is also supported by the data showing an increase in H5N1 viruses isolated from 2011 to 2012, which correlates to a switch from clade 2.2.2 to the possibly more genetically stable 2.3.2.1a, which has been shown to be found primarily in the cloaca and feces of backyard ducks.^9^

H9N2 viruses are found largely in the retail markets instead of the wholesale markets in both chicken and quail. Quail are commercially raised, handled and housed separately in retail markets from other domestic poultry in Bangladesh. The commercial rearing and LBM practices regarding quail are akin to the practices for chicken, with the exception of processing. Quail are reared for both egg and meat production, and are often purchased by restaurateurs. Although quail are typically slaughtered in the markets, meat processing takes place in restaurant kitchens. Given that quail cages in LBMs are rarely empty due to demand, and that quail is the species with the second highest prevalence for H9N2 viruses, this can explain why retail markets, in general, have a higher prevalence of H9N2 than wholesale markets. Moreover, there is a continuous supply of food and water for poultry in retail markets while wholesale markets do not supply either food or water for poultry since birds are typically sold before the end of the day. This lack of a common food and water source in wholesale markets reduce the opportunities for oral transmission of H9N2 viruses. In addition, the short length of stay in wholesale markets may also contribute to the lower prevalence seen due to the insufficient time for transmission to occur.

Previous studies^7^, ^12^ have shown that H9N2 viruses isolated from Bangladesh are reassortant viruses, possessing the nonstructural (NS), and polymerase PA and PB1 genes, from an H7N3 virus from Pakistan. H9N2 viruses donated their internal genes to several H5 and H7 viruses of public health concern.^22^ It has also been previously shown^12^ that chicken H9N2 in Bangladesh is antigenically conserved compared to quail H9N2 which shows antigenic drift. H9N2 viruses tend to evolve rapidly and acquire mutations that confer replication in mammalian hosts, much like H5N1 isolated from Bangladesh.^12^ As chicken is the more popular species for food consumption when compared to quail, it is more likely that interspecies transmission at the human–animal interface would occur between chicken and human rather than quail and human, despite the fact that IAVs are detected more frequently in quail than in other domestic poultry. Because there is no seasonal distribution detected in our surveillance for both H5N1 and H9N2 viruses, this indicates an enzootic situation that must be addressed using multidisciplinary ‘One Health' approach.

Currently, LBM practices in Dhaka do not include regular cleaning, disinfection or changing of the food and water troughs for poultry (Supplementary Figures S2–S6). Birds that were sampled in the markets came from different parts of the country, representing more than 20 farms, and were housed together in bamboo cages in only two wholesale markets, creating a breeding ground for amplification events of AIVs to occur. One of the earlier studies conducted that the identified risk factors involved in the spread of HPAI H5N1 in Hong Kong showed that LBMs are a major risk factor in the perpetuation and transmission of AIV infection.^3^ In an effort to combat the role LBMs play in the continuation, amplification and circulation of AIVs especially HPAI viruses, both Hong Kong SAR and China have been able to implement procedures that not only help reduce human exposure to these viruses, but also assist in alleviating the potential for a pandemic.^23^, ^24^, ^25^ For instance, a ban on keeping unsold poultry overnight in LBMs and routine closure for disinfection drastically reduce the risk of poultry-to-poultry and poultry-to-human transmission of AIVs.^23^, ^24^, ^25^

Because many of the vendors and poultry workers at LBMs in Bangladesh depend on revenue generated from trading poultry for their livelihood, there is no financial incentive to comply with any regulations that require a temporary shutdown of market operations. Despite the difficulties associated with temporary market closure, it is imperative from a public health perspective that infection control measures be implemented to reduce the risk of AIV transmission. A Hong Kong study showed that ‘rest days' in which markets are cleared of all poultry and cleaned can disrupt the spread of AIVs.^26^ Similarly, the intervention by Bangladeshi authorities of incorporating one ‘rest day' per month for all LBMs may assist in reducing the threat of AIV exposure to the general public. It is necessary to reiterate the importance of daily cleaning that includes but is not limited to, sanitizing contact surfaces, cleaning water troughs, and changing water daily even when temporary closures, banning overnight stay of poultry or increasing biosecurity are cost-prohibitive. Any intervention should be made after proper risk-communication and discussion with the market vendors and workers.

Although there are similarities in the perpetuation of IAVs in LBMs between Bangladesh and Hong Kong, when it comes to geographical location, levels of biosecurity, live bird market hygiene, overall culture and socioeconomic status, and poultry movement through the market system, there are even greater similarities between Bangladesh and countries in Southern Asia and Northern Africa, such as India and Egypt than between Bangladesh and Far Eastern countries such as Vietnam and China.^27^, ^28^, ^29^ Chicken is the more popular avian species among consumers in countries like India, Egypt and Bangladesh, whereas duck is the more popular avian species in countries like Hong Kong, China and Vietnam. Dietary restrictions based on religion also have some impact on not only the various species that are farmed, but also the prevalence of AIVs at the human–animal interface. With swine being regarded as the ‘mixing vessel' for the emergence of potentially novel human pandemic strains of influenza virus,^30^ it is worth noting that pig production systems in predominantly Muslim countries, such as Bangladesh and Egypt, are uncommon. Pig farming is neither lucrative nor a large-scale industry in these countries, making most of the pig production systems backyard-like in nature with little to no biosecurity. In Bangladesh, poverty and stigmatization of pig farmers make implementation of biosecurity measures and health intervention very difficult, allowing for increased chances of interspecies transmission of IAVs, especially with domestic birds.^31^

Conversely, China is the largest producer of swine in the world,^32^ and as such, there is a greater chance of zoonotic transmission at the human–animal interface and appearance of novel strains of IAVs in China than in Bangladesh.^33^, ^34^ These differences between China and Bangladesh play an important role on the prevalence of different species within the live bird markets, as well as the prevalence of various subtypes of IAVs found and what impact this may have on public health. Despite these differences, the detection rate of AIVs in Bangladesh is relatively high (24%) compared to both Egypt (5%)^35^ and China (4%),^36^ which further emphasizes the need for both continued surveillance and implementation of standard precautions against IAV transmission and infection. Accordingly, attention must be given to these factors as well as the conditions within LBMs in Bangladesh in order to better assess the appropriate preventive measures that should be taken in order to reduce the dissemination of AIVs and lessen the threat of an epidemic.

This study is not without limitations. The LBMs and farms surveyed are all located in or near Dhaka. Even though birds that enter the markets in Dhaka come from various parts of the country, the prevalence seen in these LBMs do not reflect AIV prevalence in LBMs as a whole in Bangladesh. The design of this study, which includes the choice of sampling sites, sampling methods within each site, and the number of samples collected at each site, did undergo some changes during the course of this study for various reasons. As such, data collected were not consistent throughout the 7-year period. Due to changes in funding, the total number of samples successfully isolated and subtyped decreased since the beginning of the study, and the selection strategy implemented for characterizing viruses isolated introduced sampling bias. Regardless, continued surveillance in Bangladesh is necessary in order to gather information that will provide a more in-depth understanding of the dynamics of AIVs in LBMs at the human–animal interface and their impact on public health.

## Figures and Tables

**Table 1:** Comparison of rRT-PCR influenza A positive samples by variable

Variable	Collected samples, no. (%)^a^	Influenza A-positive samples, no. (%)^b^	*P*-values^c^
*Sample type*			
Oropharyngeal	8081 (38.3%)	2689 (33.3%)	<0.001
Cloacal	4609 (21.8%)	857 (18.6%)	
Environmental (fecal)	6395 (30.3%)	861 (13.5%)	
Environmental (water)	2011 (9.5%)	653 (32.5%)	
*Species*			
Chickens	14 213 (67.4%)	3276 (23.0%)	<0.001
Ducks	3426 (16.2%)	844 (24.6%)	
Geese	58 (0.3%)	11 (19.0%)	
Pigeons	1165 (5.5%)	173 (14.8%)	
Quail	2234 (10.6%)	756 (33.8%)	
*Bird type*			
Domestic	20 958 (99.3%)	5043 (24.1%)	<0.001
Pet	138 (0.7%)	17 (12.3%)	
*Bird health status*			
Dead	8 (0.0%)	0 (0.0%)	<0.001
Healthy	20 952 (99.3%)	5055 (24.1%)	
Sick	10 (0.0%)	0 (0.0%)	
Undetermined	126 (0.6%)	5 (4.0%)	
*Domestic bird location*			
Backyard	225 (1.1%)	5 (2.2%)	<0.001
Farm	6202 (29.4%)	597 (9.6%)	
LBM	14 669 (69.5%)	4458 (30.4%)	
*Market type*			
Retail	11 137 (75.9%)	3332 (29.9%)	0.027
Wholesale	3532 (24.1%)	1126 (31.9%)	

Abbreviation: live bird market, LBM.

Influenza A positive samples by rRT-PCR and the total number of samples for each variable collected from LBMs and farms in Bangladesh from 2008 to 2016. The numbers and percentages shown under the ‘collected samples' column represent the actual number and percentage of that category out of the total number of samples for the specified variables. The numbers and percentages shown under the ‘influenza A-positive samples' column represent the actual number and percentage of that category out of the total number of samples for the specified variables. *P*-values shown are based on a statistical significance of *P*<0.05, comparing the rates of influenza A positivity across variable categories.

a Percentage of total samples collected.

b Of samples within category.

c By *χ*^2^-test comparing positive rates across variable categories.

**Table 2:** Comparison of egg isolation positive samples by subtype and variable

Variable	Positive samples, no. (%)^a^	H5	H9	Subtype^b^ H5/H9	Other	*P*-values^c^
*Sample type*						
Oropharyngeal	825 (63.4%)	48 (5.8%)	653 (79.2%)	105 (12.7%)	19 (2.3%)	<0.001
Cloacal	155 (11.9%)	18 (11.6%)	115 (74.2%)	11 (7.1%)	11 (7.1%)	
Environment (fecal)	164 (12.6%)	29 (17.7%)	115 (70.1%)	6 (3.7%)	14 (8.5%)	
Environment (water)	158 (12.1%)	13 (8.2%)	134 (84.8%)	11 (7.0%)	0 (0.0%)	
*Species*						
Chickens	1027 (78.9%)	42 (4.1%)	893 (87.0%)	92 (9.0%)	0 (0.0%)	<0.001
Ducks	128 (9.8%)	43 (33.6%)	12 (9.4%)	29 (22.7%)	44 (34.4%)	
Geese	1 (0.1%)	1 (100.0%)	0 (0.0%)	0 (0.0%)	0 (0.0%)	
Pigeons	2 (0.2%)	0 (0.0%)	2 (100.0%)	0 (0.0%)	0 (0.0%)	
Quail	144 (10.2%)	22 (15.3%)	110 (76.4%)	12 (8.3%)	0 (0.0%)	
*Bird type*						
Domestic	1300 (99.8%)	108 (8.3%)	1015 (78.1%)	133 (10.2%)	44 (3.4%)	NS
Pet	2 (0.2%)	0 (0.0%)	2 (100.0%)	0 (0.0%)	0 (0.0%)	
*Bird health status*						
Healthy	1300 (99.8%)	108 (8.3%)	1015 (78.1%)	133 (10.2%)	44 (3.4%)	NS
Undetermined	2 (0.2%)	0 (0.0%)	2 (100.0%)	0 (0.0%)	0 (0.0%)	
*Domestic bird location*						
Farm	43 (3.3%)	1 (2.3%)	11 (25.6%)	0 (0.0%)	31 (72.1%)	<0.001
LBM	1259 (96.7%)	107 (8.5%)	1006 (79.9%)	133 (10.6%)	13 (1.0%)	
*Market type*						
Retail	1015 (80.6%)	80 (7.9%)	834 (82.2%)	96 (8.5%)	5 (0.5%)	<0.001
Wholesale	244 (19.4%)	27 (25.2%)	172 (70.5%)	37 (15.2%)	8 (3.3%)	

Abbreviations: live bird market, LBM; not significant, NS.

Influenza A-positive samples by egg isolation and the subtypes for each variable collected from LBMs and farms in Bangladesh from 2008 to 2016. The numbers and percentages shown under the ‘positive samples' column represent the actual number and percentage of that category out of the total number of samples for the specified variables. The numbers and percentages shown under the ‘subtype' column represent the actual number and percentage of the specified subtypes out of the total number of samples for the specified variables. *P*-values shown are based on a statistical significance of *P*<0.05, comparing the rates of influenza A positivity across variable categories.

a Percentage of total samples positive for influenza A.

b Percentage of samples within category.

c By *χ*^2^-test comparing positive rates across variable categories.
