# Supplementary material for: Insight into live bird markets of Bangladesh: an overview of the dynamics of transmission of H5N1 and H9N2 avian influenza viruses
Source: Emerg Microbes Infect. 2017 Mar 8;6(3):e12–. doi: 10.1038/emi.2016.142 (PMC5378921; doi:10.1038/emi.2016.142)
Supplement: Supplementary Figure S2 [file emi2016142x2.pdf]

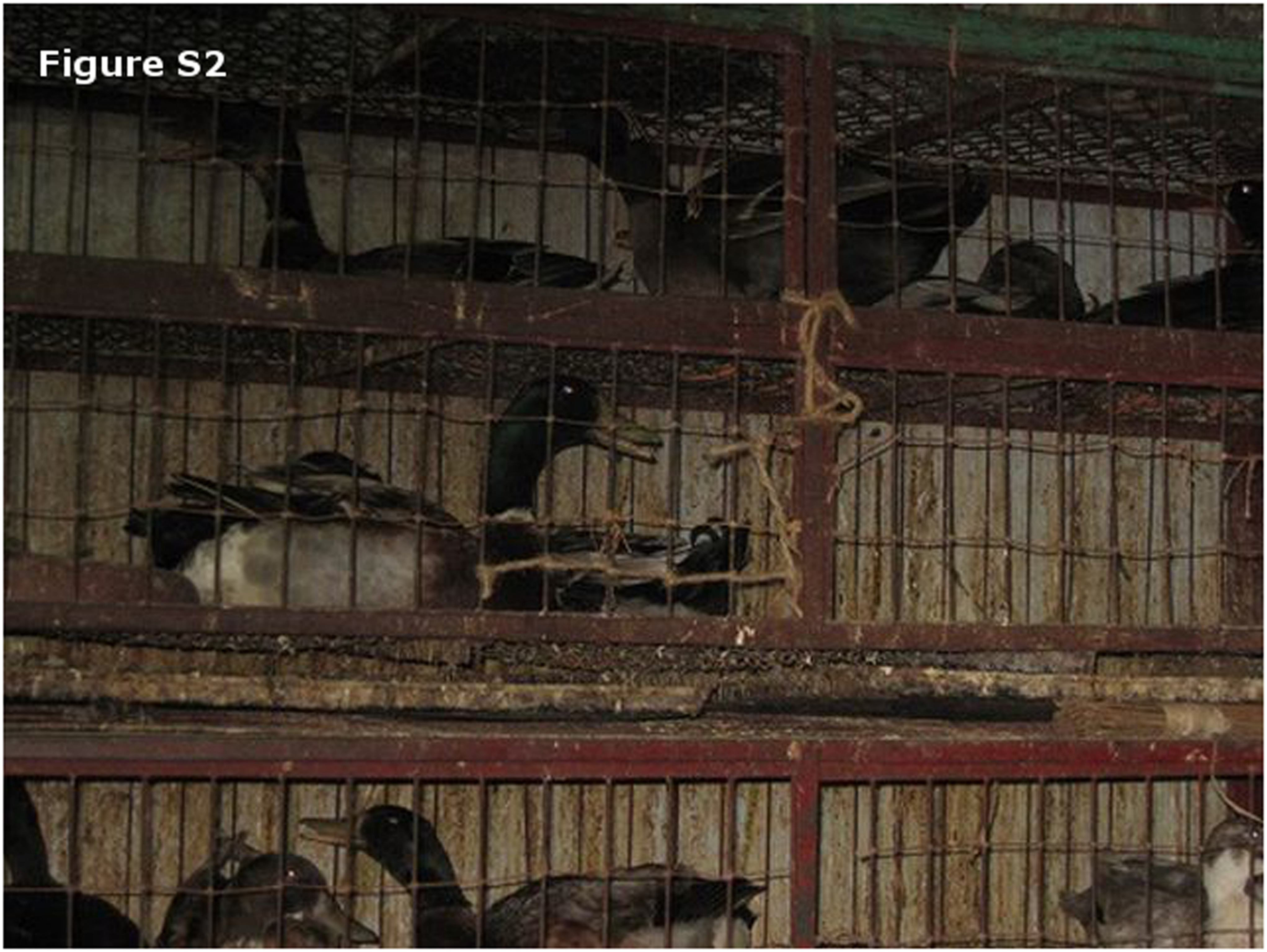

Supplementary Figure S2 Domestic ducks in the retail markets are housed in multistory wire cages, similar to those that house chickens, and have continuous access to food and water.
