# Supplementary material for: Insight into live bird markets of Bangladesh: an overview of the dynamics of transmission of H5N1 and H9N2 avian influenza viruses
Source: Emerg Microbes Infect. 2017 Mar 8;6(3):e12–. doi: 10.1038/emi.2016.142 (PMC5378921; doi:10.1038/emi.2016.142)
Supplement: Supplementary Figure S3 [file emi2016142x3.pdf]

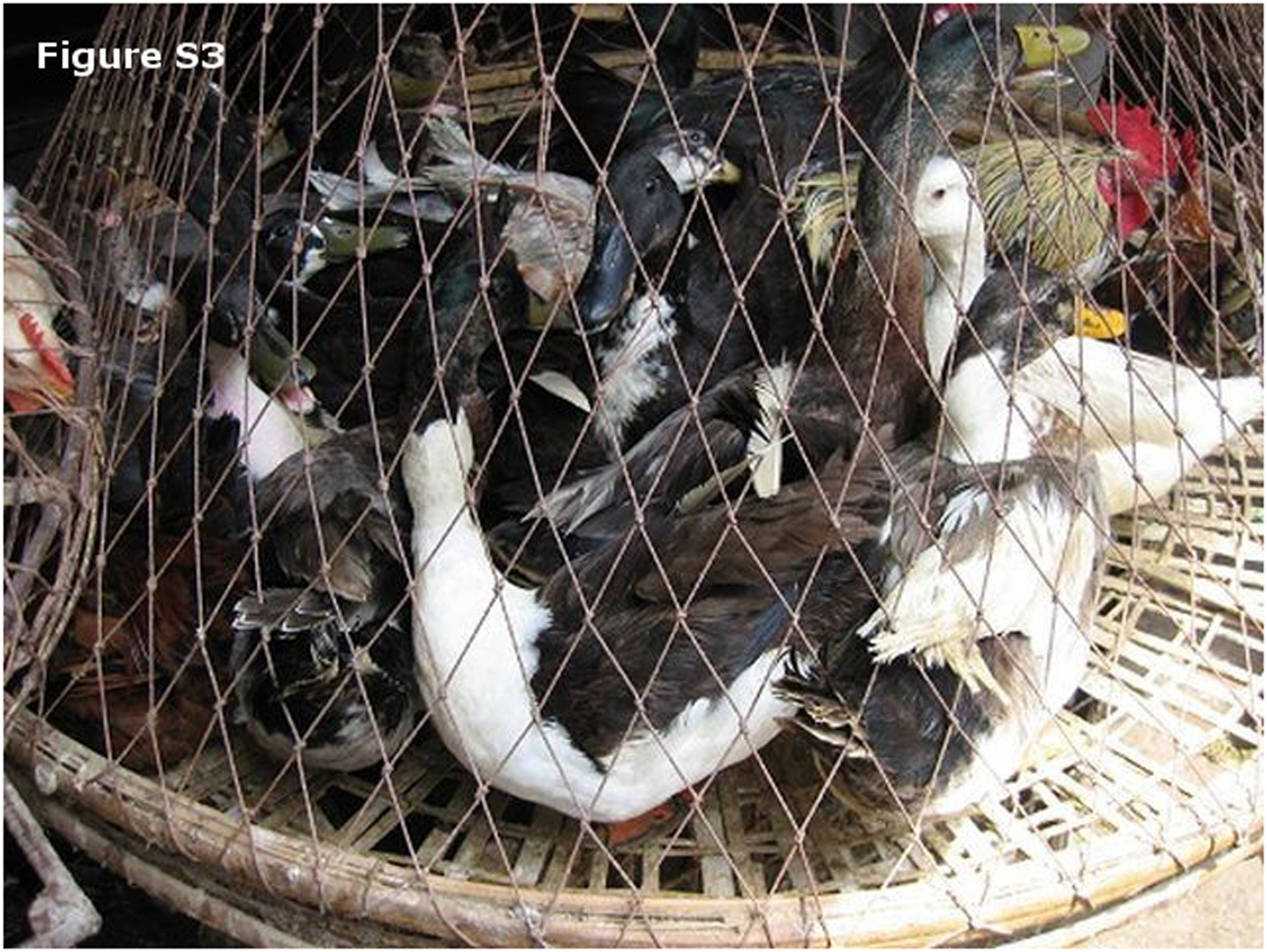

Supplementary Figure S3 Interspecies housing of ducks and chickens in bamboo cages in LBMs in Bangladesh. The cohousing of ducks and chickens can allow for interspecies transmission of AIVs in LBMs.
