# Supplementary material for: Insight into live bird markets of Bangladesh: an overview of the dynamics of transmission of H5N1 and H9N2 avian influenza viruses
Source: Emerg Microbes Infect. 2017 Mar 8;6(3):e12–. doi: 10.1038/emi.2016.142 (PMC5378921; doi:10.1038/emi.2016.142)
Supplement: Supplementary Figure S4 [file emi2016142x4.pdf]

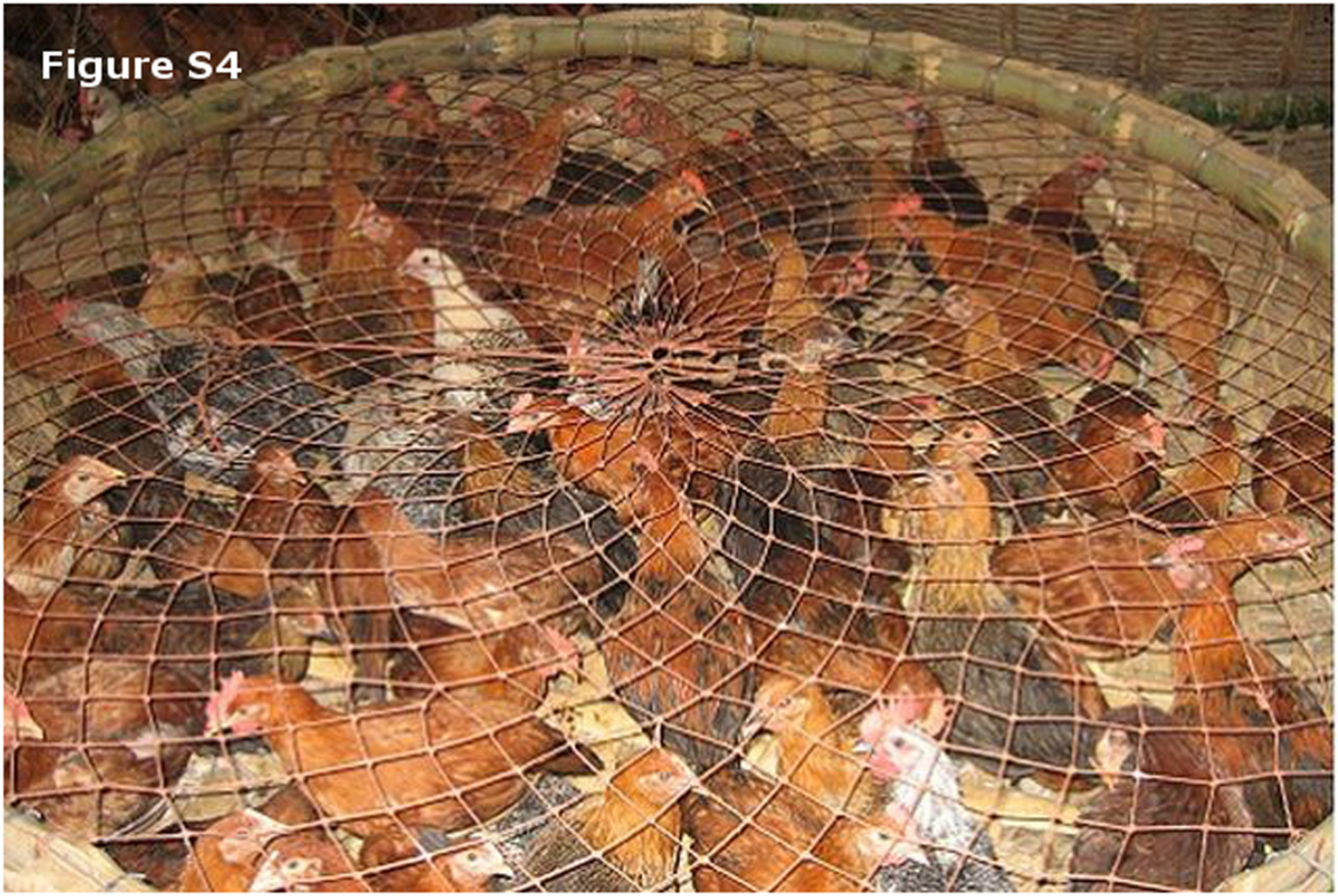

Supplementary Figure S4 Chickens in wholesale markets are usually housed in cages made of bamboo and rope as shown. The chickens that are to be distributed to retail markets typically do not have access to a common food or water source. Once chickens are moved to retail markets, they are housed in multistory wire cages with a continuous supply of food and water.
