# Supplementary material for: Insight into live bird markets of Bangladesh: an overview of the dynamics of transmission of H5N1 and H9N2 avian influenza viruses
Source: Emerg Microbes Infect. 2017 Mar 8;6(3):e12–. doi: 10.1038/emi.2016.142 (PMC5378921; doi:10.1038/emi.2016.142)
Supplement: Supplementary Figure S5 [file emi2016142x5.pdf]

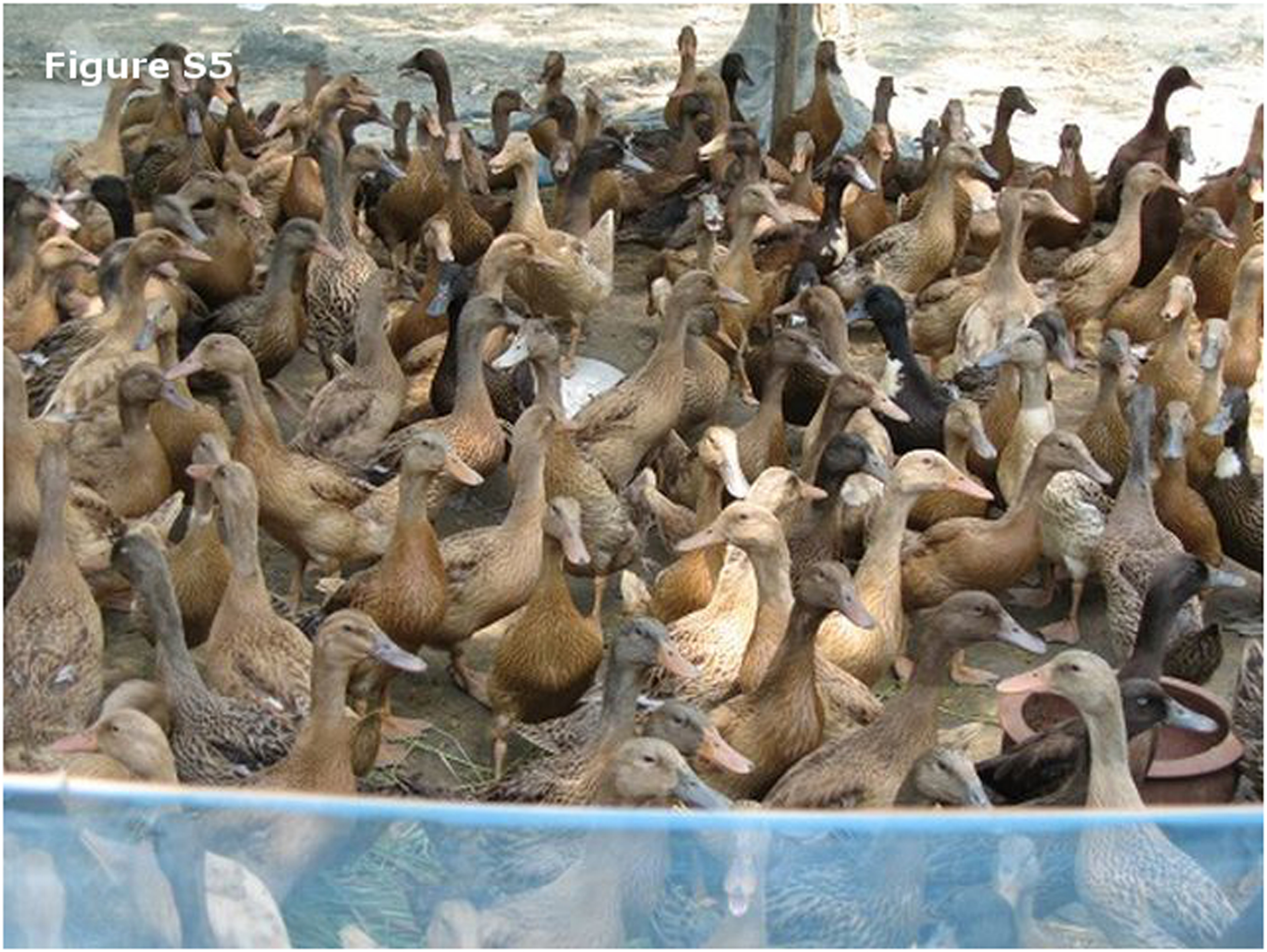

Supplementary Figure S5 Domestic ducks in Bangladesh are gathered in enclosures for brief periods during the course of the day. They are housed in these enclosures overnight and released during in the morning where they spend the bulk of the time foraging.
