# Supplementary material for: Insight into live bird markets of Bangladesh: an overview of the dynamics of transmission of H5N1 and H9N2 avian influenza viruses
Source: Emerg Microbes Infect. 2017 Mar 8;6(3):e12–. doi: 10.1038/emi.2016.142 (PMC5378921; doi:10.1038/emi.2016.142)
Supplement: Supplementary Figure S6 [file emi2016142x6.pdf]

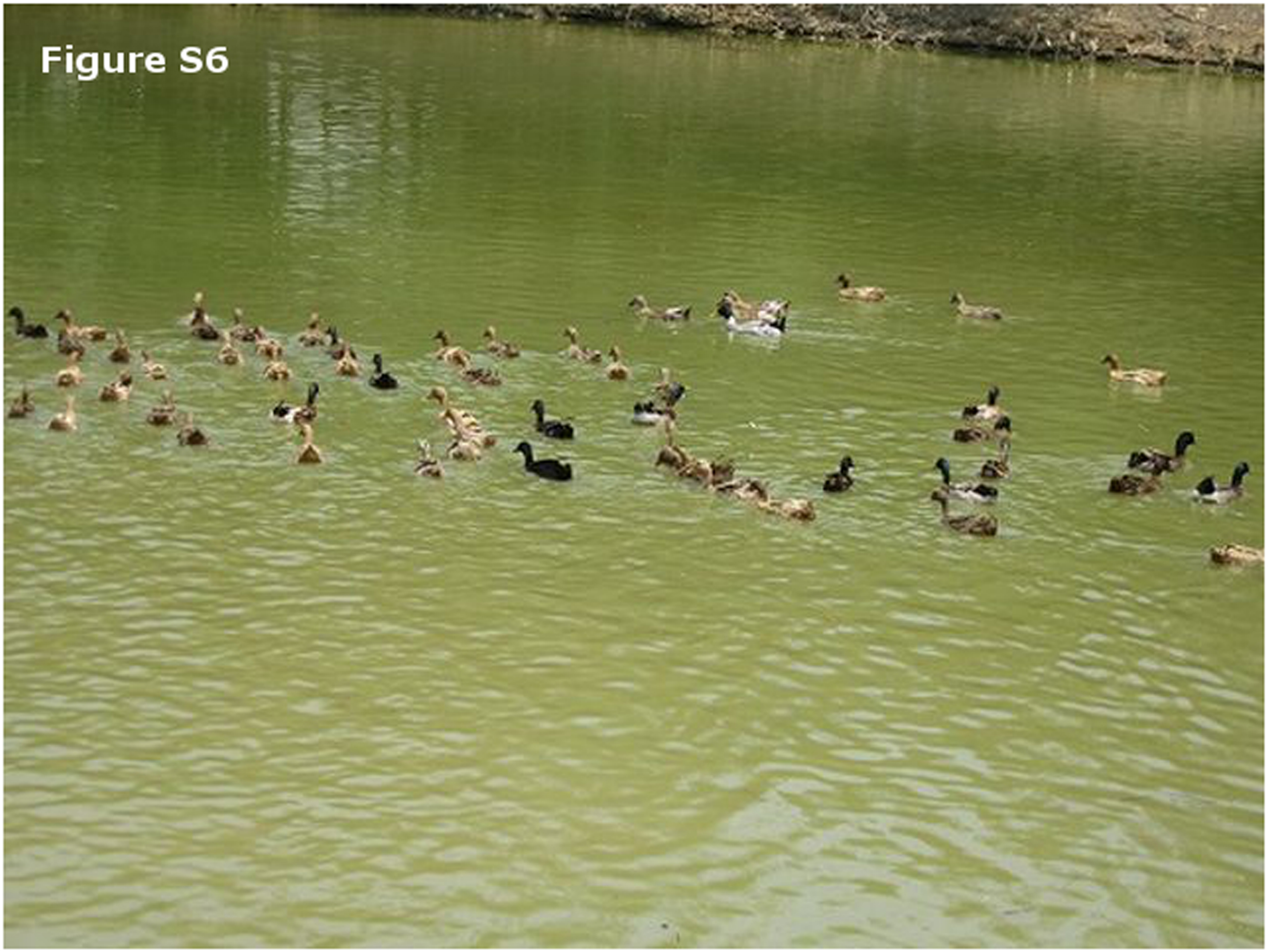

Supplementary Figure S6 Domestic ducks reside in free-range conditions on farms in Bangladesh. As shown, they are allowed to freely move about the waterways and wetlands to scavenge for food. It is here that they have interactions with wild birds and may possible come into contact with wild bird AIVs.
